# Chemistry *(Animal)*

**Published:** 1818-02

**Authors:** 


### III. CHEMISTRY (*Animal*).

1. *Analysis of the Blood.* About twelve months ago, we presented a translation of a paper by Vauquelin, *on the colouring principle of the blood of animals*,* wherein this distinguished chemist confirmed the inferences previously stated by Mr. Brande, that the colour of this fluid is not attributable to the presence of iron: and we really regarded the point as finally and satisfactorily decided by the concurrent testimony of these gentlemen. The Swedish Berzelius, however, has recently questioned the correctness of their experiments, and the legitimacy of the conclusions deduced from them; and advances opinions, diametrically opposite, on the subject. His paper † opens with an allusion to the discovery of oxide of iron in the incinerated blood by former chemists, and the various conjectures entertained by them, as to the state of combination in which the metal exists in that fluid. He then refers to the chemical analysis of the blood, published by himself, some years since, in a work on *Animal Chemistry*. It is there, he observes, proved that the colouring matter of the blood is not a solution of sub-phosphate of iron in the serum; and that neither tannin, prussiate of potash, nor any of the most delicate re-agents, detects in it the slightest trace of oxide of iron. Yet iron, he adds, is contained in the colouring matter, though incineration is necessary in order to obtain it. Half the ashes, which result from this process, is oxide of iron; but, in procuring the oxide as well as the lime and phosphates, complete incineration is requisite; for they are combined with the other substances in such a manner that they cannot be extracted, even by the strongest acids. Hence he concludes, that the iron is not combined, in a state of oxide, with the colouring matter, but that metallic iron is therein united with the other elements, in the same manner as the carbon, hydrogen, &c. Fibrine and albumen he has found, on incineration, to contain no iron.

After particularly adverting to the experiments of Messrs. Brande and Vauquelin, the Swedish chemist proposes the two following queries :—Does the colouring matter contain iron or not? and how far does the contained iron contribute to its colour? In answer to the *first*, he states his belief that he has already, in the memoir above-mentioned, advanced enough, both respecting the iron contained in the colouring matter, and the form under which it exists, to render needless any farther exposition. The process by which he heretofore obtained the substance in question, differs considerably from that employed by Vauquelin; ‡ and, even in repeating the experi-

* See *Medico-Chirurgical Journal and Review*, vol. ii. p. 508.

† *Note sur le Principe colorant du sang des Animaux.* Par M. Berzelius.—*Annales de Chimie et de Physique*, Mai 1817.

‡ The coagulum, after being drained, was cut into thin slices, and placed upon bibulous paper, in order to separate from it as much of the serum as possible. The colouring matter was then dissolved in water, ment detailed by the latter, he reports a dissimilarity of result, for which he declares himself unable to account. These experiments, it is added, however, prove that the colouring matter of the blood, even when exposed to the action of re-agents, which tend to decompose it, and dissolve the iron, yet retains this metal, which forms a constituent part of it, and may be obtained from its ashes.

The *second* query, he acknowledges, is very difficult of decision. It seems evident that the iron does not produce the colour of the blood, as was once supposed, under the form of oxide dissolved in the fluid: but its presence in the colouring matter may yet exert some influence upon the colour. The colouring matter, possessed of many of the properties of fibrine and albumen, differs only from them in colour, and in the iron which it contains. It resembles yet more perfectly the crystalline lens; and the ashes of the latter contain but slight traces of iron. Again, the black matter, which covers the choroid coat of the eye, yields red ashes, in which a great quantity of oxide of iron is contained. From these observations, Berzelius considers it sufficiently proved that the colouring matter of the blood is distinguished from colourless animal substances by the quantity of oxide of iron which it yields upon incineration; and that *probably* the iron may contribute to the deep colour of this substance. M. Vauquelin, he concludes, in his examination of the pretended discovery of M. Brande, seems not to have incinerated the colouring matter; without which, extraction of the contained iron is impracticable: and as to Mr. Brande, who has employed incineration, and yet detected traces of iron so slight as almost to elude observation, it is very difficult to explain the result which he has obtained.

Mr. Brande, we hope, will not suffer to remain thus unsettled an interesting question in animal physiology; but vindicate himself with his wonted ability and decision from the charges of inaccuracy brought against him by his Swedish antagonist.

CHEMISTRY (*Vegetable*).—1. *Analysis of Opium.* It was, some time since, our intention to have presented the British reader with a full account of Sertuerner's lately published Memoir on "Morphium and the Meconic Acid considered as essential parts of opium."* But this has now been done by one of our most respectable contemporaries,† in such a stile of accuracy and extent, as

---

which left the fibrine untouched and colourless. The colouring matter thus dissolved, was separated from the water, by evaporation, for such experiments as required it in an unaltered and soluble state; and by ebullition, which caused it to coagulate.

* *De la Morphine et de l'Acide Meconique, considérés comme parties essentielles de l'Opium.* *Annalen der Physik,* B. xxv. *Annales de Chimie et de Physique.* Mai 1817.

† See *London Medical Repository*, vol. viii. p. 433.—We beg leave to remark, nor in so doing fear we any misconstruction of our design by render our task superfluous. We shall, therefore, merely transcribe the Appendix and Conclusions of this elaborate production, as introductory to a notice of some critical remarks upon it by M. Robiquet, and of Orfila's Report on the Physiological Action of Morphium, which we propose taking in our next number.

" *Appendix*." "Thus far had I proceeded in my Memoir, when I had occasion to make the following remarks on the preparation of morphium and meconic acid.—1. Take eight ounces of powdered opium, rub it with two or three ounces of concentrated acetic acid and a little distilled water; dilute the paste with two or three pounds of cold water, and filter the liquid. In this nearly colourless solution are contained acetate and meconate of morphium, extractive, and traces of the combination of extractive with morphium. 2. Precipitate the morphium by ammonia, and evaporate the liquid to one-fourth. After cooling, separate the morphium by filtration, and precipitate the meconate of ammonia by a proper quantity of acetate of barytes. On evaporating the liquid to dryness, a little meconate of barytes is still deposited. The extract from which the acetates have been separated by concentrated alcohol, is almost pure extractive. I have taken ten grains of it without inconvenience. The residue contains the combination of extractive in excess with morphium, little soluble in water. I have successively treated it with sulphuric acid, diluted with six parts of water; and decomposed the acid solution by ammonia. This decomposition is not perfect, for there invariably remains morphium with an excess of extractive (brown meconic acid). This *acid extractive* or *brown meconic acid*, is destitute of effect: from the morphium are derived its pernicious properties."

" *Conclusions*." "Crude opium contains then *neutral extractive* and *acid extractive*, which are both without effect on the animal œconomy. The latter is combined with morphium; but it also combines with meconate of morphium, and becomes soluble in water. This combination is only in part decomposed by the water: for the residue contains traces of meconic acid; which, forming a triple combination with much morphium and extractive, dissolves but gradually in water. Hence it is that extract of opium, prepared with cold water, contains only part of the meconate of morphium, and a little of the combination of morphium with extractive. By an addition of acetic acid, this combination loses part of its morphium; and the meconate of morphium separates from the brown meconate of morphium."

---

the liberal-minded Editors of the *Repository*, that there exists, in the translation of the memoir, an uncorrected verbal error; which, although in itself trivial, throws obscurity over a paragraph. At the twenty-first line of page 436 of the *Repository*, acetate of *copper* should be substituted for "acetate of *morphium*."—Edit.

